# ST-Elevation Myocardial Infarction Complicated by Ventricular Septal Defect Is Associated With Very High Mortality Without Improvement Over Time

**DOI:** 10.31083/RCM49035

**Published:** 2026-05-13

**Authors:** Mohammad Reza Movahed, Arteen Rasti, Mehrtash Hashemzadeh

**Affiliations:** ^1^Department of Medicine, University of Arizona, Phoenix, AZ 85004, USA; ^2^Department of Medicine, University of Arizona Sarver Heart Center, Tucson, AZ 85724, USA

**Keywords:** ventricular septal defect, mechanical complications, acute coronary syndrome, acute ST-elevation myocardial infarction, acute myocardial infarction

## Abstract

**Background:**

This study aimed to examine mortality trends among patients who presented with ST-elevation myocardial infarction (STEMI) complicated by ventricular septal defect (VSD) between 2016 and 2022.

**Methods:**

Using the Nationwide Inpatient Sample (NIS) database and International Classification of Diseases (ICD)-10 codes for patients with STEMI and VSD from 2016–2022, we evaluated mortality trends over these years.

**Results:**

A total of 2735 patients with STEMI-associated VSD were identified. Total average mortality was 50.65%, with no significant average improvement over the years studied. In the multiple logistic regression analysis, the only clinical or demographic predictors of mortality were increasing age, which was associated with higher mortality (multivariate odds ratio (MVOR) 95% confidence interval (CI) = 1.05 (1.03–1.07); *p* < 0.001) and Asian race, which was associated with lower mortality (MVOR 95% CI = 0.26 (0.07–1.00); *p* = 0.05).

**Conclusions:**

STEMI complicated by VSD is associated with very high mortality (>50%) and shows no significant improvement over the study period. Furthermore, increasing age is an independent predictor of mortality, whereas Asian populations may be associated with a lower mortality risk.

## 1. Introduction

Acute myocardial infarction (MI) can occur from a multitude of factors like atherosclerosis, thrombosis, vasospasm, embolism, or dissection [1, 2]. MI is one of the major causes of death in both the United States and worldwide. Nearly three million people are affected by the disease worldwide, with over one million deaths annually in the United States alone [1]. ST-segment elevation myocardial infarction (STEMI) [1, 3] is the most serious presentation of MI. There are several mechanical complications in addition to myocardial damage that can occur during or post-MI, including but not limited to mitral regurgitation secondary to papillary muscle rupture, ventricular septal defect (VSD), true ventricular aneurysm, right ventricular infarction, pseudoaneurysm, and free wall rupture [4]. Each of these complications and others can result in cardiogenic shock, hemodynamic instability, heart failure, and arrhythmias [1, 4].

VSD is a rare (~1–2% incidence rate) but serious life-threatening complication of MI that usually occurs within the first week after the infarction [5]. Conservative treatment leads to nearly 94% mortality and 47% mortality in treatment with surgical intervention [5]. Initial management includes initial stabilization, including afterload reduction and hemodynamic stabilization with either an intra-aortic balloon pump (IABP) or other mechanical circulatory support (MCS) devices such as extracorporeal membrane oxygenation (ECMO) or Impella prior to surgery [6]. There are multiple surgical approaches to VSD closure, including infarct exclusion with the David technique followed by primary repair with the Daggett technique, patching or suturing to close the defect. Other options include modified transatrial approaches such as left atriotomy, utilization of temporary LV assist devices, percutaneous closure, or rare heart transplantation [6]. Current demographic trends for myocardial infarction include higher rates of mortality among Black individuals, men, older patients, and those in the Southern and rural United States [7]. However, there have yet to be any major studies examining mortality trends in any demographics in patients with VSD status post-STEMI. Our study aimed to identify any demographics or clinical predictors that contribute to mortality in STEMI patients with subsequent VSD.

## 2. Material and Source

### 2.1 Data Source

We utilized the National Inpatient Sample (NIS), a component of the Healthcare Cost and Utilization Project (HCUP), for our study. The NIS database approximates a 20% sample of discharges from community hospitals in the USA and contains weighted discharge information for over 35 million admissions each year. In total, this sample represents 98% of the total U.S. population [8].

### 2.2 Study Population

The NIS database for the years 2016–2022 was utilized. To generate and stratify the study population, we used the International Classification of Diseases, Tenth Revision Clinical Modification (ICD-10-CM), and Procedure Coding System (ICD-10-PCS) codes for our study. Patients with a VSD as a complication of STEMI were identified using the ICD-10-CM diagnosis code I23.2. Patients under the age of 18 were excluded as we only studied adult patients.

### 2.3 Study Outcomes

The primary outcomes were the presence of VSD and mortality rates in patients above the age of 18 using multiple logistic regression for baseline characteristics and comorbidities such as age, gender, race, diabetes, hypertension, hyperlipidemia, chronic obstructive pulmonary disease (COPD), smoking, and chronic kidney disease (CKD).

### 2.4 Statistical Analysis

Patient demographic, clinical, and hospital characteristics are reported as percentages in the tables. Odds ratios and 95% confidence intervals were reported. Categorical outcomes were assessed using Chi-squared analysis. The methods for determining risk factors for death were based on the most common cardiovascular comorbidities and baseline characteristics. All *p*-values are 2-sided and *p* < 0.05 was used as statistically significant. Data was analyzed using STATA 17 (Stata Corporation, College Station, TX, USA).

## 3. Results

**Fig. 1.**
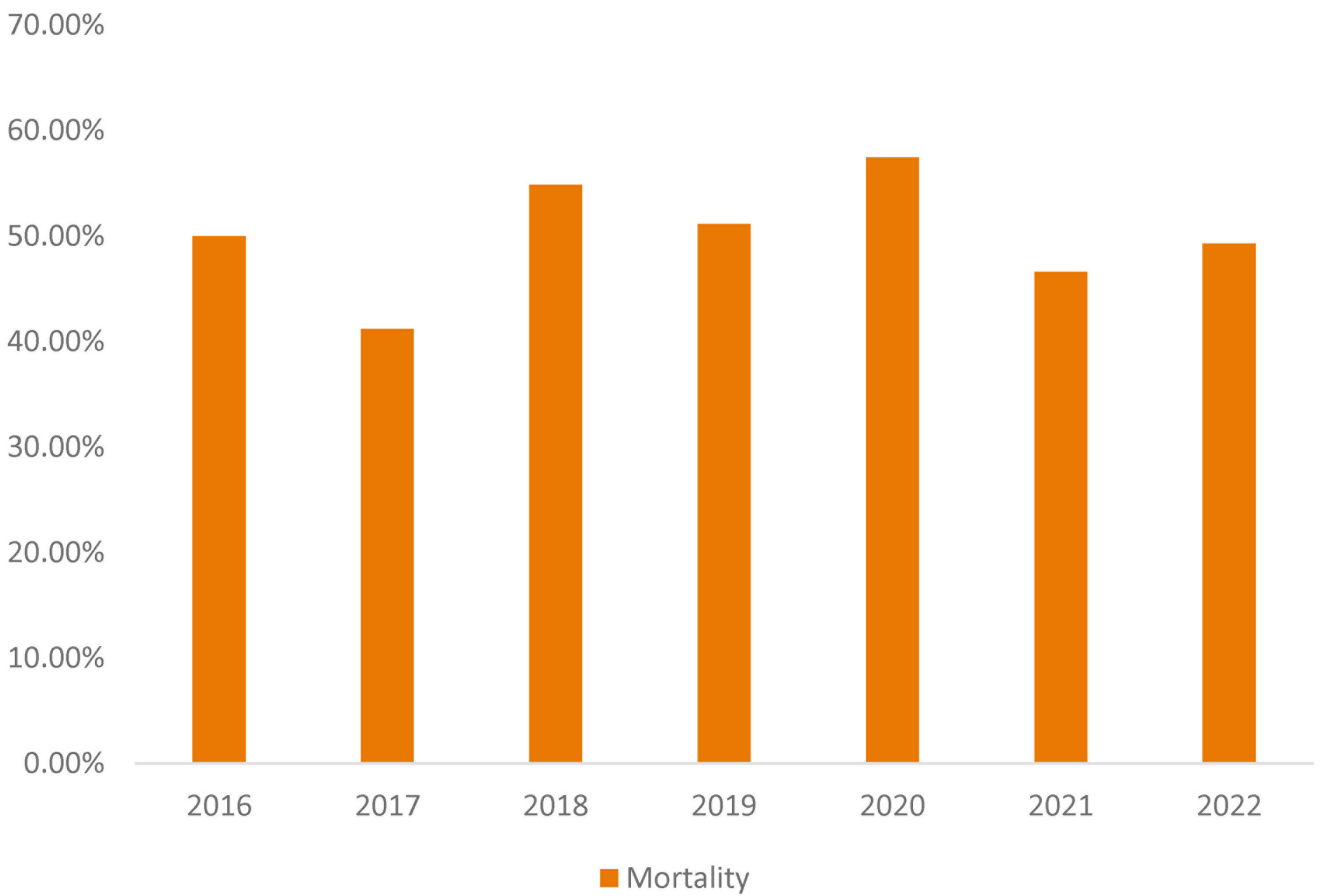
**Overall mortality rate of all ventricular septal defect (VSD) complications from ST-elevation myocardial infarction (STEMI) in patients from 2016 to 2022**.

A total of 2735 patients with STEMI-associated VSD were identified through the NIS database out of a total of 3,635,810 patients with STEMI. The total mortality for the 2735 patients with STEMI-associated VSD was 1385 deaths. The average mortality was over 50.65%, and it did not significantly improve over the years studied. 2016 had a 50% mortality rate, which improved to 41.18% in 2017 before worsening to 54.84%, 51.14%, and 57.45% in the years 2018–2020, respectively. Mortality dropped in the years 2021 and 2022 to 46.58% and 49.28%, respectively (Fig. 1). There were no clinical or demographic predictors for mortality from the single analysis, except for higher mortality with increasing age and lower mortality in the Asian race (Table 1). Multivariate odds ratio for each year of age: (multivariate odds ratio (MVOR) 95% confidence interval (CI) = 1.05 (1.03–1.07), *p* < 0.001). Multivariate odds ratio for Asian race: (MVOR 95% CI = 0.26 (0.07–1.00), *p* = 0.05).

**Table 1.** **Multivariate predictors of all patients who died from ST-elevation myocardial infarction associated with ventricular septal defect**.

Mortality only in STEMI-VSD patients		*p*-value	MVOR	95% CI for MVOR	
				Lower	Upper
Diabetes		0.44	0.85	0.57	1.28
Hypertension		0.48	0.85	0.55	1.33
Hyperlipidemia		0.85	0.96	0.65	1.43
COPD		0.11	1.67	0.9	3.11
Smoking		0.22	0.76	0.48	1.19
CKD		0.28	0.78	0.5	1.22
Age		<0.001	1.05	1.03	1.07
Gender					
	Male		REF		
	Female	0.26	1.25	0.84	1.86
Race					
	White		REF		
	Black	0.71	0.81	0.27	2.44
	Hispanic	0.64	1.19	0.58	2.47
	Asian/Pac Isl	0.05	0.26	0.07	1.00
	Native American	0.67	0.62	0.07	5.64
	Others	0.18	1.96	0.73	5.30

MVOR, multivariate odds ratio; CI, confidence interval; COPD, chronic obstructive pulmonary disease; CKD, chronic kidney disease; Pac Isl, Pacific Islander.

## 4. Discussion

The incidence rate (~0.08%) for VSD post-STEMI from the years 2016–2022 from the NIS data was lower than the generally reported incidence rate of 1–2% [5]. The mortality rate of 50.65% was similar to the general mortality rate of 51% with surgical treatment [5], and it is unlikely that the standard of treatment has changed during the seven years of the study. The increased rates of mortality in the year 2020 (57.45%) compared to previous years, other than 2018, may be attributed to the COVID-19 pandemic, which is supported by the noted decrease in the years 2021 and 2022. Studies have shown that although the overall incidence of hospital admissions for STEMI decreased by 8.2%, patients with COVID-19 and STEMI suffered from a mortality rate of 45.2% compared to 10.7% admitted for STEMI without COVID-19 co-infection [9]. A recent study done by Nasso *et al*. [10] mentioned increased prevalence of post-MI VSD, which they suggested was due to their hospital during the pandemic, though mortality rates were similar when treated appropriately. Incidence of VSD was seen to have increased during this time due to patients presenting to the hospital late out of fear of being exposed to COVID-19 [10]. Further studies are needed to establish a causal trend between COVID-19 and VSD status-post STEMI. While previous studies have shown that Black Americans and men are at an increased risk for acute MI and have higher rates of mortality [7], it is suggestable that this is unlikely due to the significantly lower mortality (MVOR 95% CI = 0.81 (0.27–2.44), *p* = 0.71) and no significant difference of VSD mortality in gender (prevalence: MVOR 95% CI = 1.07 (0.90–1.27), *p* = 0.449) (mortality: MVOR 95% CI = 1.25 (0.84–1.86), *p* = 0.26).

Findings from the univariate analysis showed increasing mortality with age (MVOR 95% CI = 1.05 (1.03–1.07), *p* < 0.001) and lower mortality in the Asian race (MVOR 95% CI = 0.26 (0.07–1.00), *p* = 0.05). The findings of increased mortality with age are expected and consistent with previous studies of age being associated with increased mortality status post-MI [7] and suggest that VSD may play a contributing factor. However, it is also important to note that overall mortality rates, regardless of etiology, will generally increase with age. Multiple previous studies in Asian populations have shown an increased risk of mortality after acute MI [11, 12, 13]. A particular study conducted by Sakaguchi *et al*. [14] found that mortality outcomes of surgical repair of post-MI VSD in a Japanese cohort were 24.3% overall and 33.0% intra-operatively. Reasons for increased mortality in Asian populations after myocardial infarctions include higher prevalence of comorbidities like diabetes, hypertension, and kidney disease, as well as delayed presentation and intervention [14]. It is also important to consider that South Asian patients present with STEMI at younger age than their white counterparts (51.6 years vs 62.7 years), which may cause superior healing and collagen deposition of myocardial tissue, thus possibly improving outcomes after VSD formation [13]. The findings from our study should be followed up to investigate the reasoning for the unique lower mortality rate in the Asian population from VSD status-post STEMI, as there is no current literature that suggests Asian populations have lower mortality; rather, the opposite.

## 5. Limitations

There are several limitations to our study. ICD-10 coding has inherent limitations, and VSD status-post MI may not have been reported as such in patient charts. NIS can also be limited in attributing specific complications of a condition, such as STEMI, and can only include data for admitted patients. NIS data does not provide critical information such as time from symptom onset to hospital presentation, history of prior ischemic heart disease, previous cardiomyopathy or heart failure, and relevant comorbidities that may have contraindicated surgical intervention. Furthermore, our NIS data does not show specific initial management (such as surgical repair, percutaneous intervention, or conservative management), hospital stay, or other factors that may impact on these patients and their outcomes. The findings from our study warrant further investigation to discern further correlation and reasoning for the unique results obtained. In addition, suggestions for future studies include expanding the sample duration beyond seven years and incorporating NSTEMIs in addition to STEMIs. Due to the large number of MI complications that may have an overlapping impact on patient mortality, generating a future multivariate analysis for the same demographic variables with other post-myocardial complications (papillary muscle rupture, ventricular aneurysm, ventricular free-wall rupture, etc.) should also be considered.

## 6. Conclusions

STEMI complicated with VSD is associated with very high mortality rates, especially in patients unable to receive surgical treatment. From our large in-patient database, increasing age is an independent predictor of mortality status-post STEMI-associated VSD, with lower mortality rates being seen overall in Asian populations. Our study highlights the need for further research in identifying patient trends for catastrophic VSD, possibly to help develop future criteria for surgical or mechanical interventions.

## Availability of Data and Materials

Data is publicly available upon purchase from NIS website.
